# Reduction of selenite to Se(0) nanoparticles by filamentous bacterium *Streptomyces* sp. ES2-5 isolated from a selenium mining soil

**DOI:** 10.1186/s12934-016-0554-z

**Published:** 2016-09-15

**Authors:** Yuanqing Tan, Rong Yao, Rui Wang, Dan Wang, Gejiao Wang, Shixue Zheng

**Affiliations:** State Key Laboratory of Agricultural Microbiology, College of Life Science and Technology, Huazhong Agricultural University, Wuhan, 430070 People’s Republic of China

**Keywords:** Actinobacteria, Glutathione, Selenium nanoparticles (SeNPs), Intracellular deposition, Export system, Aerobe

## Abstract

**Background:**

Selenium (Se) is an essential trace element in living systems. Microorganisms play a pivotal role in the selenium cycle both in life and in environment. Different bacterial strains are able to reduce Se(IV) (selenite) and (or) Se(VI) (selenate) to less toxic Se(0) with the formation of Se nanoparticles (SeNPs). The biogenic SeNPs have exhibited promising application prospects in medicine, biosensors and environmental remediation. These microorganisms might be explored as potential biofactories for synthesis of metal(loid) nanoparticles.

**Results:**

A strictly aerobic, branched actinomycete strain, ES2-5, was isolated from a selenium mining soil in southwest China, identified as *Streptomyces* sp. based on 16S rRNA gene sequence, physiologic and morphologic characteristics. Both SEM and TEM-EDX analysis showed that Se(IV) was reduced to Se(0) with the formation of SeNPs as a linear chain in the cytoplasm. The sizes of the SeNPs were in the range of 50–500 nm. The cellular concentration of glutathione per biomass decreased along with Se(IV) reduction, and no SeNPs were observed in different sub-cellular fractions in presence of NADPH or NADH as an electron donor, indicating glutathione is most possibly involved in vivo Se(IV) reduction. Strain ES2-5 was resistant to some heavy metal(loid)s such as Se(IV), Cr(VI) and Zn(II) with minimal inhibitory concentration of 50, 80 and 1.5 mM, respectively.

**Conclusions:**

The reducing mechanism of Se(IV) to elemental SeNPs under aerobic condition was investigated in a filamentous strain of *Streptomyces*. Se(IV) reduction is mediated by glutathione and then SeNPs synthesis happens inside of the cells. The SeNPs are released via hypha lysis or fragmentation. It would be very useful in Se bioremediation if *Streptomyces* sp. ES2-5 is applied to the contaminated site because of its ability of spore reproduction, Se(IV) reduction, and adaptation in soil.

## Background

Selenium (Se) is an essential trace element for the adequate and healthy life of human, animal, bacterium and other living systems and has an uneven distribution in the Earth’s crust [1]. Today, selenium is well recognized to play fundamental roles on several physiological functions in diverse organisms, such as biosynthesis of selenocysteine (Sec), the 21st amino acid with specific UGA stop codon, and many selenoenzymes including formate dehydrogenase, thioredoxin reductase, and glutathione peroxidase [2–4]. In human, either Se excess or deficiency results in more than 20 kinds of symptoms such as growth retardation, endemic diseases, impaired bone metabolism and risk of diabetes [5]. Events of selenium toxicity in human have been reported in Enshi, Hubei province of China and in Indian Punjab [6]. Therefore, selenium contamination requires bioremediation initiatives especially in those geographic locations. Phylogenetically diverse microorganisms are involved in the transformation of selenium from one oxidation state to another and thus play a pivotal role on the selenium biogeochemical cycle [4, 7, 8]. Numerous bacteria are able to reduce the toxically soluble forms of Se(VI)/Se(IV) to less-toxic insoluble Se(0), visible as red-colored nanoparticles (SeNPs) [4, 9–14]. The biosynthesized SeNPs have been found applications in various fields including medicine as antimicrobial, antioxidant and anticancer agents [15–18], biosensors [19, 20] and environmental remediation [21–23].

Se(IV)-reducing bacteria generate SeNPs under aerobic and anaerobic conditions. Anaerobic Se(IV)-reducing bacteria encompassed many species such as *Thauera selenatis* [24], *Aeromonas salmonicida* [25], purple non-sulfur bacteria [11] and *Shewanella oneidensis* MR-1 [26]. Aerobic Se(IV)-reducing bacteria included diverse species such as *Rhizobium* sp. B1 [12], *Stenotrophomonas maltophilia* SeITE02 [27], *Pseudomonas seleniipraecipitans* CA5 [28], *Duganella* sp. and *Agrobacterium* sp. [13], *Comamonas testosteroni* S44 [29] and *Bacillus mycoides* [30]. Therefore, the most Se(IV)-reducing bacteria were distributed in alpha-, beta-, gamma-, delta-proteobacteria and Firmicutes.

Selenium nanoparticles were formed not only under aerobic and anaerobic conditions, but also appeared in the cytoplasm, periplasm and/or outside the cells in different bacteria [4, 9, 10, 13, 14, 24, 29, 31], implying the various mechanisms of Se(IV)-reduction in diverse microbes. One of mechanisms linking redox precipitation of both elemental sulfur and elemental selenium was observed outside sulfate-reducing bacterial cells [32]. The intracellular Se(IV) reduction was usually driven by reduced thiols such as glutathione (GSH) via the Painter reaction in *Rhodospirillum rubrum*, *Escherichia coli* and *Bacillus mycoides* [28, 30, 33, 34]. Moreover, diverse enzymes were responsible for Se(IV) reduction to SeNPs. The periplasmic nitrite reductase was involved in Se(IV) reduction in *T. selenatis* [24] and *Rhizobium selenitireducens* [31], while fumarate reductase catalyzed Se(IV) reduction in *Shewanella oneidensis* [26]. In addition, glutathione reductase and thioredoxin reductase in *Pseudomonas seleniipraecipitans* [28], arsenate reductase in *Bacillus selenitireducens* [35] and hydrogenase in *Clostridium pasteurianum* [36] were potentially involved in Se(IV) reduction. However, so far no gene product or enzyme solely responsible for Se(IV) reduction in aerobic bacteria has been identified in vivo.

In addition, the efflux system by which Se(0) or SeNPs deposits were exported from inside the cells to the extracellular environment still remains unknown. It was suggested that SeNPs were released into the medium via a rapid expulsion process [21] or elemental Se(0) was transported out of the cell where the SeNPs were formed [29]. The large sizes of SeNPs were also possibly released by cell lysis [37] or vesicular expulsion [9].

In this study, we isolated a filamentous actinobacterium ES2-5 from a selenium mining soil in Enshi, Hubei province of China. The process of Se(IV) reduction leading to biosynthesized SeNPs under aerobic condition was investigated using scanning electron microscopy (SEM), transmission electron microscopy (TEM) and electron dispersion spectroscopy (EDX). Evidences were provided for the SeNPs formation to be mainly in the cytoplasm of cells and then released through hyphal lysis or fragmentation. The possible mechanism of Se(IV) reduction was also proposed.

## Results

### Characteristics and taxonomic identification of the strain ES2-5

Strain ES2-5 was isolated from a selenium mine soil in Hubei province, China. The acidic soil (pH 4.7) had 38 mg kg^−1^ of total Se content and 119 mg kg^−1^ of total Cr content. Accordingly, the resistance of strain ES2-5 to Se(IV), Cr(VI) and other heavy metals was determined in 1/10 TSA plates. The minimal inhibitory concentrations (MIC) of Se(IV) and Cr(VI) were 50 and 80 mM, respectively. In contrast, the MICs of Zn(II) (1.5 mM), Cu(II) (0.2 mM), As(III) (0.05 mM) and Sb(III) (0.08 mM) were lower than that of Se(IV) and Cr(VI). In addition, strain ES2-5 has the ability to produce lecithinase and H_2_S when it grew in TSB. It was positive for motility and hemolytic reaction, whereas it was negative for utilization of citrate and hydrolysis of gelation and tyrosine.

**Fig. 1:**
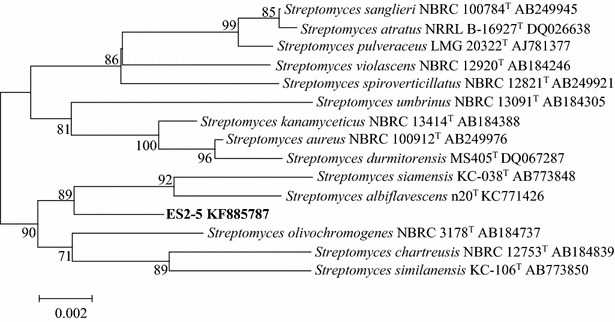
Neighbor-joining phylogenetic tree based on 16S rRNA gene sequences using MEGA software version 5, showing the phylogenetic relationship of strain ES2-5 and related type strains. Bootstrap values >50 % based on 1000 replications are shown at branch nodes. *Bar*, 0.002 substitutions per nucleotide position

**Fig. 2:**
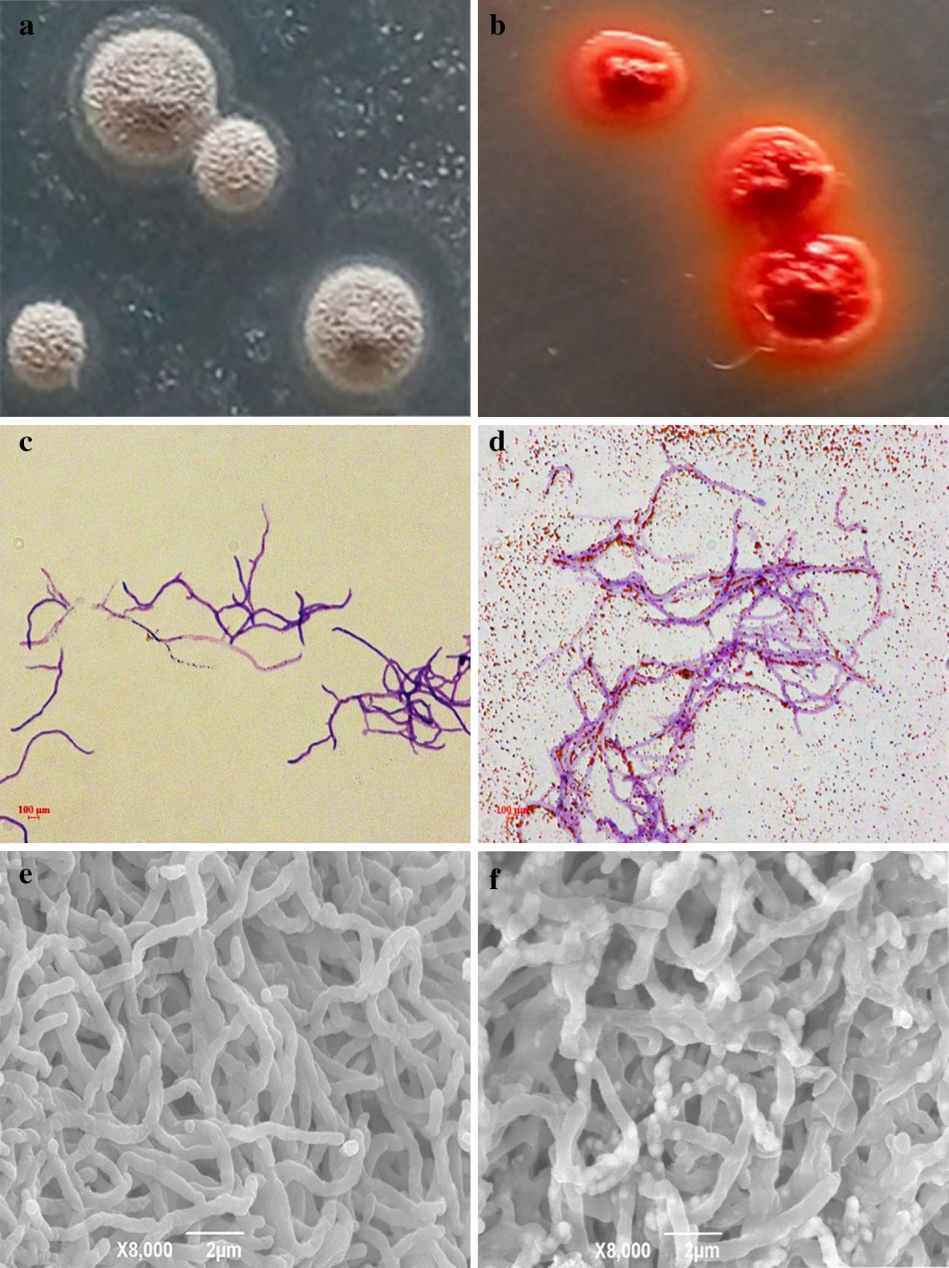
*Streptomyces* sp. ES2-5 reduced selenite to *red* elemental SeNPs. Growth of *Streptomyces* sp. ES2-5 on 1/10 TSA plates with 10.0 mM sodium selenite (**b**). LM image of SeNPs (**d**) on 1/10 TSA plates with 10.0 mM sodium selenite. SEM image of SeNPs (**f**) in 1/10 TSB broth amended with 1.0 mM sodium selenite. The** a**,** c** and** e** are control, respectively

The 16S rRNA gene sequence of strain ES2-5 (1487 bp) revealed highest similarities to that of *Streptomyces siamensis* KC-038^T^ (98.77 %), *S. kanamyceticus* NBRC 13414^T^ (98.63 %), *S. olivochromogenes* NBRC 3178^T^ (98.63 %), *S*. *aureus* NBRC 100912^T^ (98.56 %), *S. spirocerticilatus* NBRC 12821^T^ (98.43 %) and *S. albiflavescens* n20^T^ (98.39 %). Phylogenetic analyze using the neighbor-joining method showed that strain ES2-5 fell in the same cluster with *S. siamensis* KC-038^T^ (AB773848) and *S. albiflavescens* n20^T^ (KC771426C) (Fig. 1). Moreover, strain ES2-5 formed grey, floury colonies on 1/10 TSA plates (Fig. 2a), with well growing substrate mycelia, aerial hypha and sporophores. Consequently, strain ES2-5 was characterized as *Streptomyces* sp. based on the phylogenetic, morphologic and some physiologic characteristics.

### Filamentous *Streptomyces* sp. ES2-5 was able to reduce Se(IV) to SeNPs under aerobic condition

**Fig. 3:**
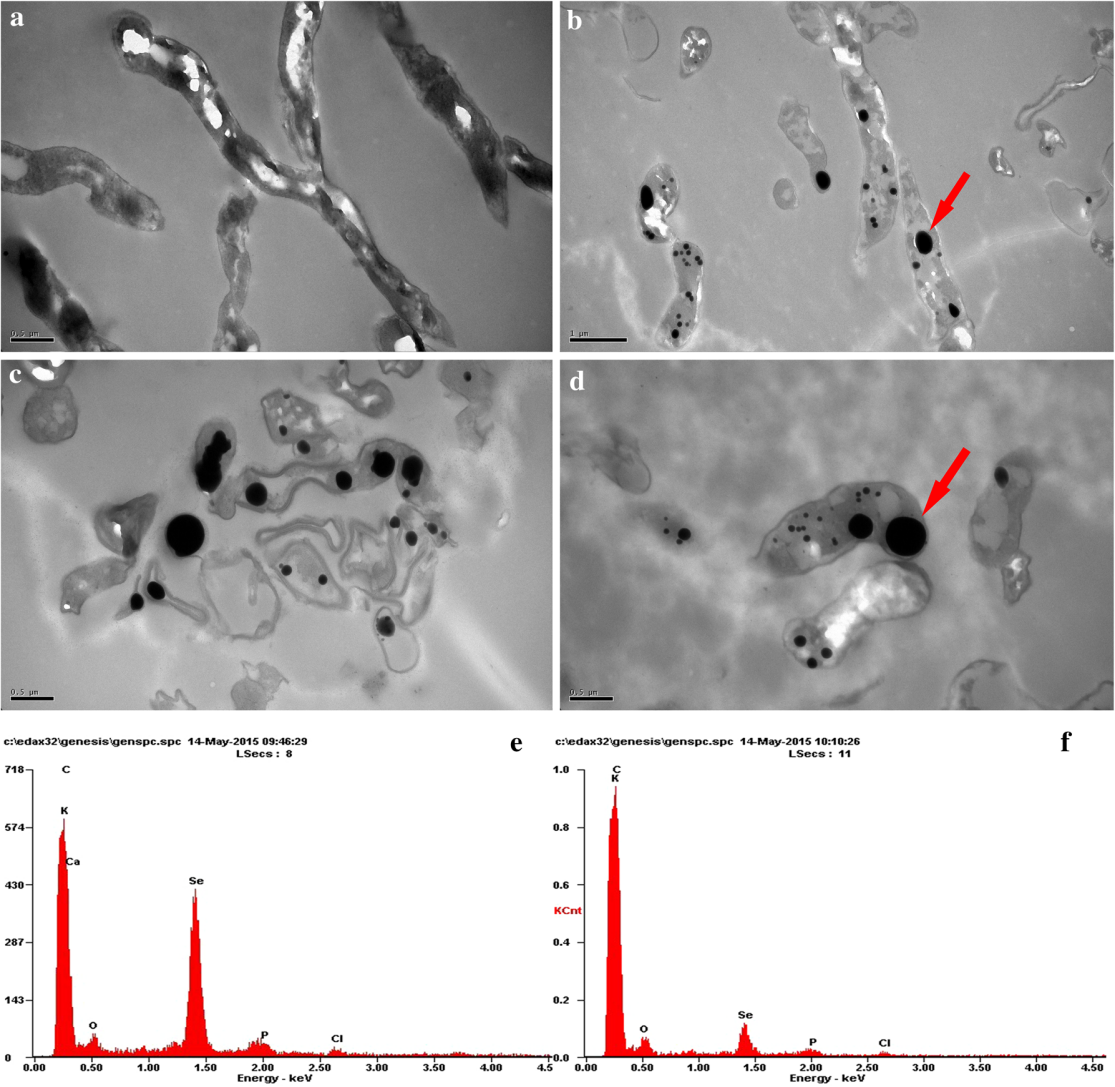
TEM micrographs and EDX spectra of *Streptomyces* sp. ES2-5 cultures grown in presence of 1.0 mM sodium selenite. **a** control; **b**-**d** intracellular Se(0) particles pointed out by arrows; **e**, **f** the emission lines for selenium are shown at 1.37 keV (peak SeLα)

*Streptomyces* sp. ES2-5 was not able to grow under anaerobic condition, indicating it is an obligate aerobe. *Streptomyces* sp. ES2-5 formed reddish colonies after 7 day’s incubation on 1/10 TSA plates amended with 10.0 mM selenite (Fig. 2b). The stained mycelia were observed in situ by light microscopy after 3 day’s incubation, the red-colored selenium particles were scattered away from mycelia or distributed as bean chains attaching on the mycelial surface (Fig. 2d). After 3 day’s incubation in 1/10 TSB broth, the mycelia were harvested and observed by SEM. Surprisingly, the selenium particles did not attach on the surface of mycelia but located in the mycelia as mature beans in pods (Fig. 2f). TEM of ultra-thin sections also revealed the common presence of intracellular Se(0) particles when mycelia were grown on Se(IV) (Fig. 3b–d). It was clear that the sizes of intracellular SeNPs varied from 50 to 500 nm and small SeNPs may aggregate into bigger particles. Dark, fine-grained nanoparticles were observed by EDX spectra which indicated that these nanoparticles were composed entirely of selenium as the expected emission peaks for selenium at 1.37 (Fig. 3e, f), 11.22 and 12.49 keV (data not shown) corresponding to the SeLα, SeKα, and SeKβ transitions, respectively, but EDX peaks for C, K, O, P, Cl and Ca were also produced, suggesting that these elements were in cytoplasm of cells.

**Fig. 4:**
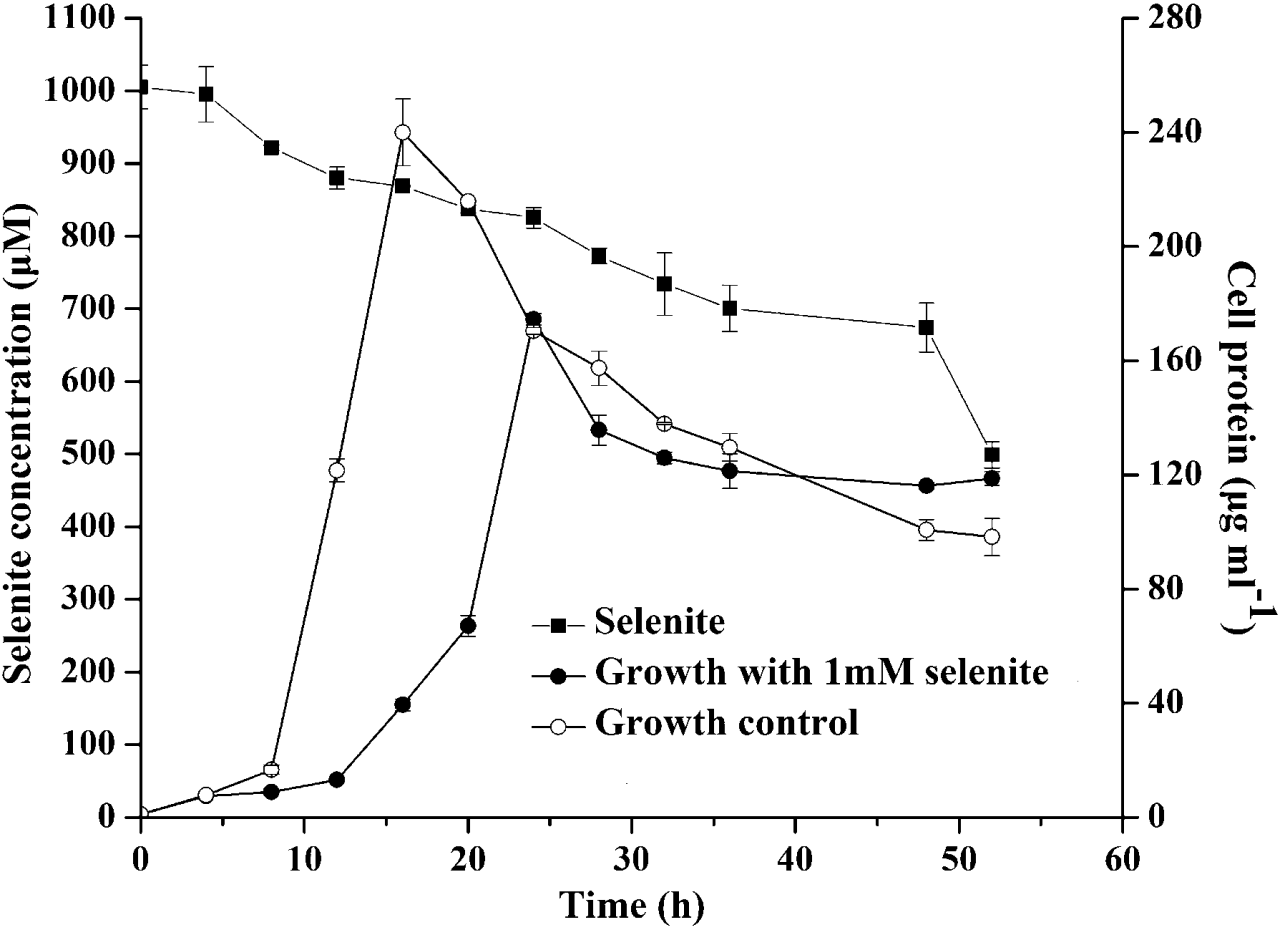
Growth and Se(IV)-reduction of *Streptomyces* sp. ES2-5 in 1/10 TSB broth at 1.0 mM concentration of selenite

The growth and capability of *Streptomyces* sp. ES2-5 to transform selenite to elemental selenium were tested in 1/10 TSB broth with the addition of 1.0 mM selenite (Fig. 4). On selenite-exposed cultures the growth was delayed with respect to controls, but after 24 h the biomass decreased gradually in a similar way. The formation of red cell suspension of elemental selenium started after 16 h of exposure to selenite. *Streptomyces* sp. ES2-5 was unable to reduce Se(IV) to elemental selenium completely. It was only able to reduce 1.0 mM Se(IV) to 0.5 mM slowly and smoothly during 52 h incubation in 1/10 TSB broth under aerobic condition.

### Mechanism of Se(IV) reduction to Se(0) nanoparticles by Filamentous *Streptomyces* sp. ES2-5

To help understand how Se(IV) is reduced, the ability of vitro Se(IV) reduction by cultural supernatant and different cellular fractions was determined. When cultural supernatant without cells was mixed with Se(IV), the reduction of Se(IV) to red-colored precipitation and decrease of Se(IV) concentrations were not observed, indicating the reduction of Se(IV) is processed in cells. Moreover, neither red-colored precipitation nor decrease of Se(IV) concentrations appeared in the cytoplasmic fraction or in cell membrane fraction with NADPH or NADH as electron donors. These results suggest that NADPH or NADH dependent reductase and reduced chemicals are not involved in vitro Se(IV) reduction. Consequently, the concentrations of glutathione (GSH) per biomass in cells (intracellular) and in cultural broth (extracellular) were determined when *Streptomyces* sp. ES2-5 grew in 1/10 TSB broth at 1.0 mM concentration of selenite.

**Fig. 5:**
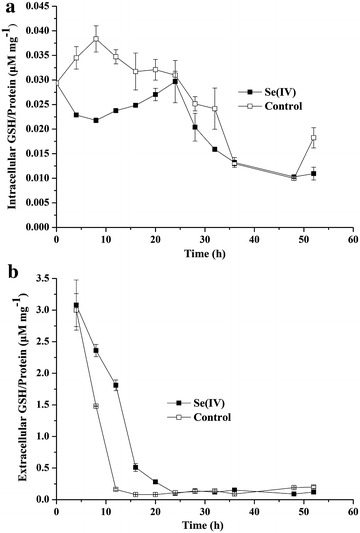
The intracellular glutathione per biomass **a** and extracellular glutathione per biomass **b** of *Streptomyces* sp. ES2-5 in 1/10 TSB at 1.0 mM concentration of selenite

In selenite-exposed cultures the intracellular GSH content per biomass was lower than in controls during first 24 h of incubation (Fig. 5a). While, the extracellular GSH content showed an opposite pattern (Fig. 5b). After 24 h the intercellular and extracellular GSH contents in selenite-exposed cultures were similar to controls.

## Discussion

Although the reduction of Se oxyanions to Se(0) nanoparticles by microorganisms has been known for some time [4, 9, 10, 13, 14, 24, 29, 38, 39], the SeNP-synthetic process and Se(IV)-reducing mechanism of filamentous bacteria have not been examined previously. In this case we found a typical actinomycete, *Streptomyces* sp. ES2-5, has ability to reduce Se(IV) to Se(0) and forms SeNPs in cells. TEM and EDX analyses showed that red-colored SeNPs accumulated in the hyphae with a diameter range of 50–500 nm. These bigger particles were aggregated by small SeNPs and then arranged along with hyphal cytoplasm as particle chains (Figs. 2, 3). It does not seem possible that these large Se(0) particles in the cytoplasm could have been derived from primary cytoplasmic synthesis and met cellular assimilation. Such a system for reduction of Se(VI) to Se(0) would be a detoxification mechanism. This mechanism could result in an incomplete selenite reduction under oxic growth conditions during a limited time frame (Fig. 4), which is consistent with a previous study in *C. testosteroni* [29]. Moreover, the large Se(0) particle chains could be extremely unmatched for hyphae, and thus the particle chains should be released only upon hyphal lysis or fragmentation (Fig. 2d, f). This could be very easy for filamentous bacteria due to the hyphal extending and branched growth. Similarly, the release of large Se(0) particles from cytoplasm via cell lysis can be observed in single-celled bacteria such as *Bacillus mycoides* [30] and *B. selenitireducens* [10]. In comparison with single-celled bacteria, it seems that *Streptomyces* sp. ES2-5 was lack of the mechanism of SeNP size control. In most cases, the diameters of SeNPs were <300 nm [4]. The size of SeNPs was about 200 nm even in filamentous fungi [40]. In *Streptomyces* sp. ES2-5, the size of larger SeNPs reached 500 nm (Fig. 3). The large Se(0) particles may also been formed by the aggregation of small particles during the movement of the cytoplasmic flow, which cannot be processed in single-celled bacteria and in eukaryotic cells with functional zoning.

As the diverse reducing mechanisms of Se oxyanions, it is very different from the reduction of As (such as *ars* cluster) and S (*dsr* cluster) associated with a certain enzymatic system in various bacteria [8, 41]. There could be multiple Se-reducing pathways in a strain, e.g., at least two selenite reductases in *P. seleniipraecipitans* or in *R. selenitireducens* [28, 31]. In *Shewanella oneidensis* MR-1, only 60 % selenite was reduced by reductase FccA [26], suggesting that more pathways are responsible for Se(IV) reduction in a bacterial strain. Among these reducing determinants, reduced thiols could be involved in Se(IV) reduction at more or less extent. Three bacterial groups produce thiols encompassing glutathione (GSH) in proteobacteria, bacillithiol (BSH) in Firmicutes and mycothiol (MSH) in actinobacteria [42]. As a result, it is not surprising that the most Se reducing bacteria distributed in these thiols-rich groups. Although a few studies on Se(IV) reduction in actinobacteria [38, 39], more Se(IV)-reducing actinobacteria would be examined in the future.

In this study, no red-colored precipitation was observed in different sub-cellular fractions in presence of NADPH or NADH as electron donors, suggesting NADPH or NADH dependent Se(IV) reductase was not responsible for Se(IV) reduction compared with previous studies [29–31]. Moreover, the intracellular concentration of GSH (could be MSH, an analog of GSH) per biomass decreased with Se(IV) reduction (Fig. 5), i.e., the reduced intracellular GSH was consumed to reduce Se(IV). In contrast, there were more extracellular thiols in broth amended with selenite than in control (Fig. 5b), indicating extracellular GSH was not involved in Se(IV) reduction because of its oxidized state under aerobic condition. Accordingly, the actinobacteria specific MSH (analog of GSH) was possibly involved in Se reduction in *Streptomyces* sp. ES2-5. Although the red-colored Se(0) nanoparticles are confirmed by Se(IV) reduction, this fact does not exclude the possibility that there are additional reduced products such as selenides because *Streptomyces* sp. ES2-5 has the ability to produce H_2_S. Se(IV) or Se(0) also might be reduced to Se(-II) via metabolic pathway of H_2_S production.

Many aerobic Se-oxyanions reducing bacteria were isolated from Enshi where soil has a high content of selenium [43–45]. Diversely aerobic Se-oxyanions reducing bacteria were also collected from different terrestrial soils [4, 13, 14, 29, 30]. This is different from the aquatic environments where anaerobic bacteria are responsible for Se(VI)/Se(IV) reduction. In anaerobic bacteria, Se(VI)/Se(IV) reduction is able to process on the surface of cells which is similar to Fe(III)’s reduction in *S. oneidensis* MR-1 [26]. In contrast, it is a great challenge in aerobic bacteria to reduce Se-oxyanions on surface of cells due to oxygen prior to accept the electrons than Se(IV), or other reducing determinants under oxidized stress in extracellular environment. Consequently, reduction is mainly processed in cells and then Se(0)/SeNP is exported or released by cell lysis (in this case).

Recent studies showed SeNPs synthesized by *Streptomyces* spp. had the anticancer activity [38, 39]. It implies the potential application of SeNPs from actinobacteria. In addition, bioremediation of contaminated soils needs aerobic microbes and anaerobic microbes. *Streptomyces* sp. ES2-5 not only has ability to reduce soluble Se(IV) into insoluble and less toxic SeNPs and to produce branched hyphae and countless spores, but also can adapt to selenite or chromate contaminated condition. Its ability of reproduction and adaptation in soil would be useful in Se/Cr bioremediation if *Streptomyces* sp. ES2-5 was applied to the contaminated site together with other aerobic and anaerobic Se(IV)-reducing bacteria.

## Conclusions

A filamentous bacterium *Streptomyces* sp. was involved in reduction of Se(IV) to elemental SeNPs arranged in linear chains in cells under aerobic condition. The synthetic process of SeNPs and mechanism of Se(IV) reduction were proposed. The sizes of intracellular SeNPs varied from 50 to 500 nm and small SeNPs may aggregate into bigger particles. The cellular concentrations of GSH per biomass decreased along with Se(IV) reduction and Se(IV)-reduction did not occur in different sub-cellular fractions, showing that Se(IV) reduction was most possibly mediated by GSH in the cytoplasm, and thus the SeNPs were released via cell lysis or fragmentation.

## Methods

### The isolation, morphological and partial biochemical characters of strain ES2-5

The strain ES2-5 was isolated from a selenium mine soil (30°17′54″ N, 109°28′16″ E) with 38 mg kg^−1^ of total Se content in Hubei province, China and by serial dilutions of the sample on 1/10 TSA (tryptic soy agar, pH 7.3, Difco) containing 1 mM sodium selenite. After 2 days of incubation at 28 °C, colonies that developed a reddish color on the initial isolation plates were transferred to fresh media for further isolation, research and storage.

Strain ES2-5 was inoculated in 1/10 TSA plates supplemented with 10 mM sodium selenite. The plates without sodium selenite were served as controls. Then, the medium in plates was inserted with sterile glass slides at a diagonal angle and placed at 28 °C. After 3 days of cultivation, the cultures on the glass slide were fixed and stained using crystal violet for 2 min and then washed by water. After air-dried, the samples were observed using light microscopy.

Lecithinase enzyme activity, motility, hemolytic reaction, anaerobic growth, utilization of citrate, hydrolysis of gelation and tyrosine were tested using the conventional method. Production of H_2_S was tested according to the method described in [46].

## 16S rRNA gene sequencing and phylogenetic tree construction

The nearly-full 16S rRNA gene sequence of strain ES2-5 was amplified using 16S rDNA universal primers 27F and 1492R following the genomic DNA extraction. The accurate sequence of PCR product was acquired by sequencing after T-A cloning with a pGEM-T Easy vector (Promega). The 16S rRNA gene sequence was compared with sequences available in the EzTaxon-e server [47], and aligned with its close relatives using the CLUSTAL_X program [48]. Neighbor-joining tree was reconstructed using MEGA version 5.0 software [49]. Distances were calculated based on Kimura’s two-parameter method [50] and bootstrap analysis was performed according to 1000 resamplings [51]. The 16S rRNA gene sequence was registered as accession KF885787 in the GenBank database.

### Growth, selenite resistance and reduction, and glutathione (GSH) determination

In order to determine the minimal inhibitory concentrations (MICs), strain ES2-5 was inoculated in 1/10 TSA plates with different concentrations of Se(IV) (0, 1, 5, 10, 20, 50, 100, 150 mM) and Cr(VI), Zn(II), Cu(II), As(III) and Sb(III) at 28 °C.

The growth curve was determined by inoculating strain ES2-5 into 100 ml 1/10 TSB broth supplemented without or with 1.0 mM sodium selenite at 28 °C with shaking at 160 rpm. Cultures were taken at 4 h intervals and centrifuged at 6000×*g*, 5 min. The supernatants (a) were used to determine concentrations of extracellular GSH and selenite. The pellet was washed twice with phosphate buffer saline (PBS, pH 7.2) and re-suspended in the same buffer. Then, the suspension was sonicated for 3 min and centrifuged at 6000×*g* and 4 °C for 5 min. The supernatants (b) were collected and used to measure the concentrations of intracellular GSH and totally cellular proteins. The determination for the content of GSH was performed by using a fluorescence-based method as described in [52]. The detailed process was realized as follows. 10 mM naphthalene-2,3-dicarboxaldehyde (NDA) was dissolved in dimethylsulfoxide (DMSO) and 50 mM Tris–HCl (pH 10.0). The NDA reagent reacts with amino and sulfhydryl groups of GSH to form an adduct, which can be measured by fluorescence signal (λ exc at 472 nM and λ em at 528 nM). The standard curve of the relationship between concentrations of GSH and the value of fluorescence signal was measured by using 100, 200, 300, 400 and 500 nM reduced GSH standard solution. The GSH concentrations of samples were calculated according to standard curve and detected fluorescence value. The biomass of strain ES2-5 was tested by measuring the contents of total cellular proteins of samples using Coomassie brilliant blue G-250 method with Bovine Serum Albumin (BSA) as standard [53]. Selenite concentrations in the supernatants (a) were measured by HPLC-HG-AFS (Beijing Tian Instruments Co., Ltd., China) [54].

### Selenite reduction activity assays in cultural supernatant and cellular fractions

In order to determine the ability of vitro Se(IV) reduction by cultural supernatant and different cellular fractions, the culture was grown to log phase and centrifuged at 6000×*g*, 5 min. The cultural supernatant was collected and filtered by a filtration with 0.2 µm disks. The pellet was washed twice with phosphate buffer saline (PBS, pH 7.2) and resuspended in the same buffer for sonication. After sonication for 3 min, the cell lysate was centrifuged at 6000×*g* for 5 min to remove the cell debris. Then, the soluble supernatant was centrifuged at 20,000×*g* for 60 min to separate the cytoplasmic fraction and membrane fraction. Selenite reductase activity was determined using the following reaction mixture [29, 30]: cultural supernatant, cytoplasmic or membrane fraction; sodium selenite (final concentration 0.2 mM); NADPH or NADH (final concentration 0.2 mM). The reaction mixture was incubated at 28 °C for 24 h. Reaction mixture without addition of cultural supernatant, cytoplasmic or membrane fraction served as controls. Selenite concentrations in the reaction mixture were measured by HPLC-HG-AFS (Beijing Titan Instruments Co., Ltd., China) [54].

### Scanning electron microscopy (SEM)

Strain ES2-5 was grown in TSB supplemented without or with 1.0 mM sodium selenite at 28 °C, 160 rpm. After 3 days of cultivation, cells were centrifuged (6000 rpm, 10 min, 4 °C) and scanning electron microscopic observation was performed on the processed samples. Samples processing involves washing, fixing and drying of cells. Harvested cells were washed thrice with phosphate buffer saline (PBS, pH 8.0). Fixation was conducted with 2.5 % glutaraldehyde (24 h, 4 °C). Cells were washed again with PBS. Fixed cells were dehydrated through a series of alcohol dehydration steps (30, 50, 70, 80, 90 and 100 %) and finally freeze dried and sputter coated. The samples were then viewed using SEM (JSM-6390 JEOL JAPAN). Samples collected from the culture without addition of selenite were regarded as controls.

### Transmission electron microscopy (TEM) and SeNP analysis with energy dispersive X-ray (EDX)

To obtain ultra-thin sections for TEM and EDX analysis, harvested cells through above-mentioned method were fixed using 2 % v/v glutaraldehyde in 0.05 M sodium phosphate buffer (pH 7.2) for 24 h and were then rinsed three times in 0.15 M sodium cacodylated buffer (pH 7.2) for 2 h. The specimens were dehydrated in graded series of ethanol (70, 96 and 100 %) transferred to propylene oxide and embedded in Epon according to standard procedures. The sections, approximately 80 nm thick, were cut with an ultrathin E (Reichert Jung) microtome and collected on copper grids with Formvar supporting membranes. The sections were stained with uranyl acetate and lead citrate and then TEM-EDX (JEM2100F JAPAN) was performed.

## Abbreviations

EDX: electron dispersion spectroscopy
MIC: minimal inhibitory concentration
SEM: scanning electron microscopy
SeNPs: selenium nanoparticles
TEM: transmission electron microscopy
